# Greenness and whiteness appraisal for bioanalysis of quetiapine, levodopa and carbidopa in spiked human plasma by high performance thin layer chromatography

**DOI:** 10.1186/s13065-024-01309-w

**Published:** 2024-10-21

**Authors:** Finan T. Hindam, Amal M. Abou Al Alamein, Reham M. Arafa, Neven Ahmed, Basma M. Eltanany

**Affiliations:** 1 Egyptian Drug Authority, P. O. Box 29, Giza, Egypt; 2 Pharmaceutical Analytical Chemistry Department, Faculty of Pharmacy, Cairo University, P. O. Box 11562, Cairo, Egypt

**Keywords:** High performance thin layer chromatography, Quetiapine, Levodopa, Carbidopa, Dopamine, Spiked human plasma, Whiteness, Greenness

## Abstract

A sustainable HPTLC-densitometric method was developed for quantitative determination of Quetiapine (QUET), Levodopa (LD) and Carbidopa (CD) in presence of Dopamine (DOP) as an internal standard. This applicable technique was achieved by spiking human plasma and extraction was performed using the protein precipitation approach. The mobile phase used was acetone, dichloromethane, n-butanol, glacial acetic acid and water (3: 2.5: 2: 2: 1.75, by volume). Method validation was done according to US-FDA guidelines and was able to quantify Quetiapine, Levodopa and Carbidopa in the ranges of 100–4000, 200–8000 and 30–1300 ng/mL, respectively. Bioanalytical method validation parameters were assessed for the studied drugs. Finally**,** the analytical suggested methodology was evaluated using various green and white analytical chemistry metrics and other tools, such as the green solvent selection tool, analytical eco-scale, green analytical procedure index, analytical greenness metric approach and the red–green–blue algorithm tool. The results revealed that the applied analytical method had a minor impact on the environment and is a relatively greener option than other previously reported chromatographic methods.

**Graphical Abstract:**

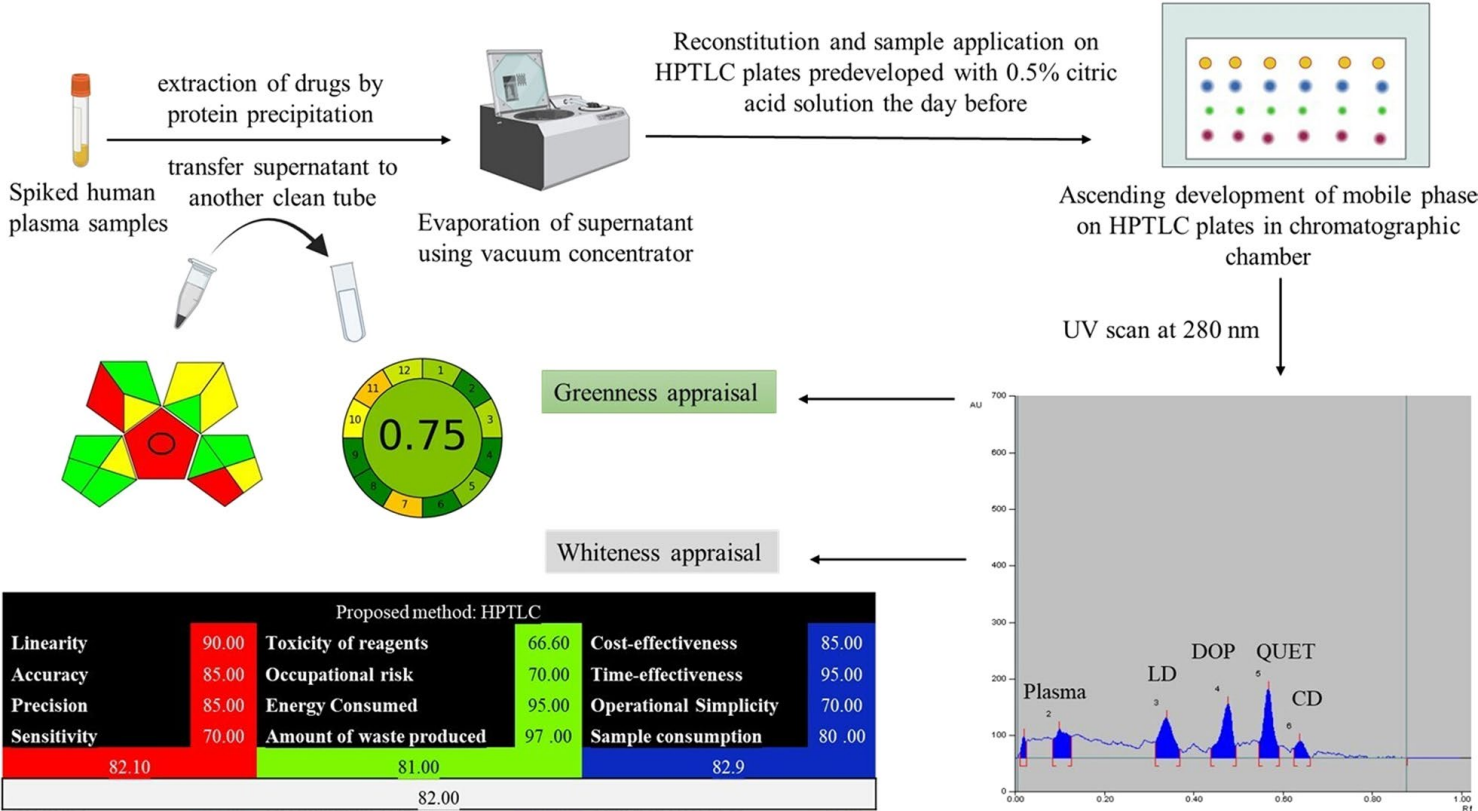

**Supplementary Information:**

The online version contains supplementary material available at 10.1186/s13065-024-01309-w.

## Introduction

Harmful chemicals, hazardous waste and other pollutants could significantly impact the ecosystem and subsequently human health. In the recent decade, Green Analytical Chemistry (GAC) has received great attention to encourage the concept of sustainable development, with an emphasis on assessing the effects that dangerous chemicals and hazardous waste have on the environment. Furthermore, White Analytical Chemistry (WAC) evolved as a supportive concept to GAC. The latter approach highlights the importance of the level of greenness of the developed analytical method as well as its performance with regard to accuracy, precision, linearity and other parameters [1–5].

It is challenging to develop an analytical procedure which retains both concepts greenness and analytical performance. Greenness assessment tools have been employed herein to determine the level to which the developed analytical method is eco-friendly and exerts a minimal impact on both human health and environment. These assessment tools include: green solvent selection tool [6], analytical eco-Scale [7], Green Analytical Procedure Index (GAPI) [8] and Analytical Greenness Metric Approach (AGREE) [9]. On the other hand, Red–Green–Blue (RGB) algorithm model [10] was applied to assess whiteness by calculating different aspects besides eco-friendliness. These aspects comprise: analytical performance, analysis cost, analysis time and others.

Parkinson’s disease psychosis (PDP) is considered one of the major complications, affecting up to 60% of patients suffering from Parkinson’s disease (PD) [11]. Psychosis is a non-motor symptom which includes delusions, hallucinations, illusions or a false sense of presence. These previously mentioned symptoms certainly have a negative impact on patients’ quality of life as they raise the demand for nursing home placement and hence increase healthcare costs [12].

Typical antipsychotic drugs block receptors of dopamine (DOP) in brain which exacerbates motor symptoms in Parkinson’s patients [11]. On the other hand, atypical antipsychotic drugs, also known as “second generation antipsychotics”, are more preferably used for treatment of PDP as they possess higher serotonergic profiles with a minimum affinity to block DOP receptors. Quetiapine (QUET) fumarate, chemically known as 2-[2-(4-Dibenzo[b,f][1,4]thiazepin-11-yl-1-piperazinyl) ethoxy] ethanol fumarate, is recognized as atypical antipsychotic drug. It has been broadly used Off-Label for management of PDP and is relatively taken at lower doses than those taken for treatment of schizophrenia or other indications [13–15].

**Fig. 1:**
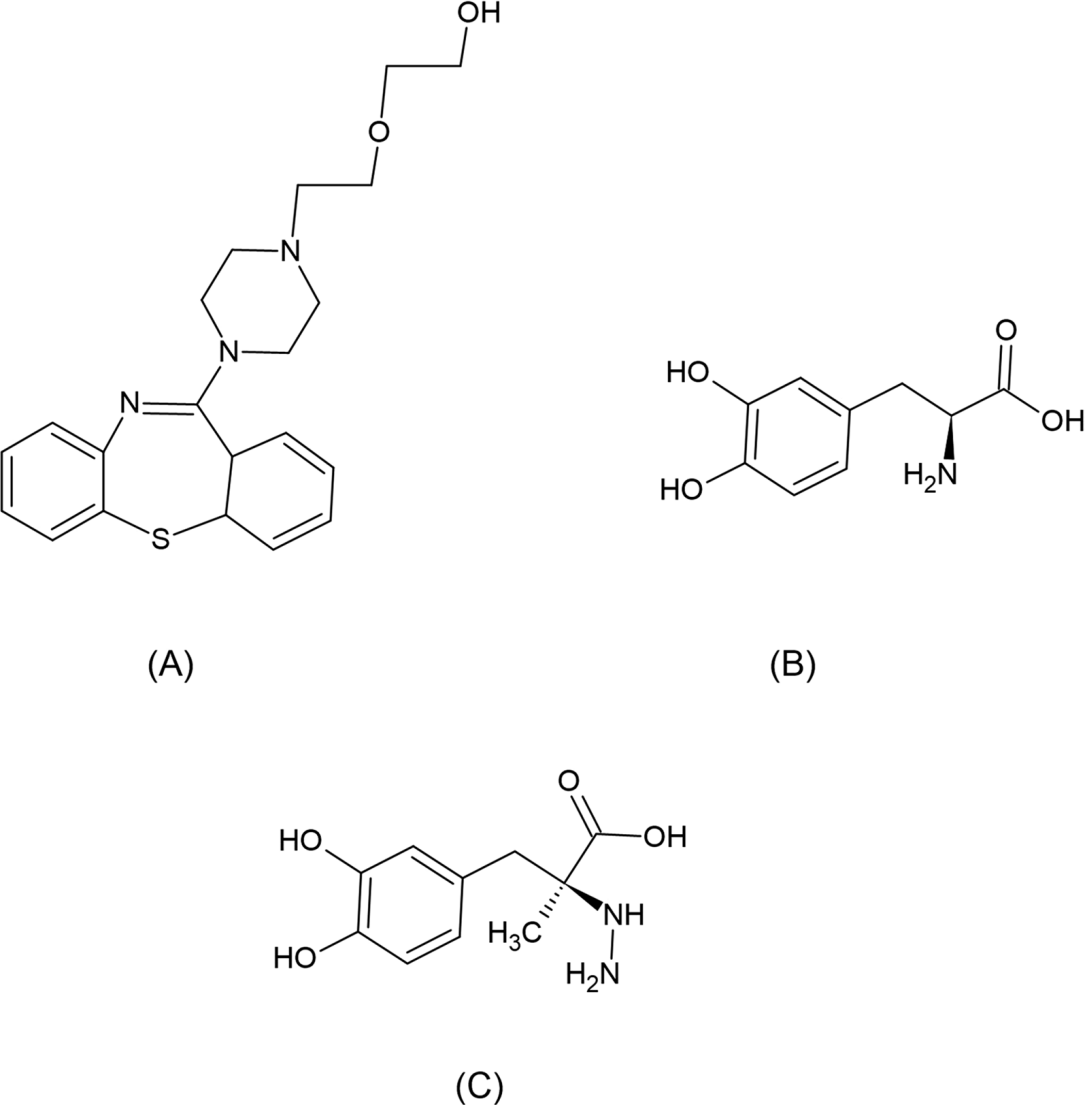
Chemical structures of the three drugs: (**A**) QUET, (**B**) LD and (**C**) CD

Levodopa (LD), referred to chemically as (–)-3-(3,4-Dihydroxyphenyl)-l-alanine, is considered a first line treatment for Parkinson’s patients [16]. LD acts as DOP precursor since it is converted to DOP after passing blood–brain barrier. One major limitation is the drug’s susceptibility to metabolism in peripheral tissues before penetration blood–brain barrier. For this reason, Carbidopa (CD) is always co-formulated with LD. CD is known chemically as (–)-l-α-Hydrazino-3,4-dihydroxy-α-methyl hydro cinnamic acid monohydrate. It acts as dopa decarboxylase inhibitor, thus preventing decarboxylation of LD to DOP in peripheral tissues. DOP, also known chemically as 4-(2-Aminoethyl) pyrocatechol hydrochloride, was used in this proposed method as an internal standard (IS). This provides an additional benefit of lack of interference with the other three studied drugs. Chemical structures for QUET, LD and CD are displayed in Fig. 1.

QUET was quantitatively determined officially through United States Pharmacopeia (USP) [17]. It is also found in literature either individually or in the presence of related metabolites, co-administered drugs, or potential impurities using the following analytical techniques: High Performance Liquid Chromatography (HPLC) [18–23], Liquid Chromatography with tandem mass spectrometry (LC–MS/MS) [24], Gas Chromatography with tandem mass spectrometry (GC–MS/MS) [25], Thin Layer Chromatography (TLC) [20, 26, 27], spectrophotometry [28, 29], fluorescence spectroscopy [30, 31], capillary zone electrophoresis (CZE) [28, 32], and electrochemical methods [33–35].

On the other hand, LD and CD were estimated using USP official methods [17] and quantitatively analyzed using various analytical methods, either alone or in combination with other co-administered drugs or related metabolites, such as HPLC [36–42], LC–MS/MS [43, 44], TLC [45–47], spectrophotometry [48–50], fluorescence spectroscopy [51], CZE [52], and electrochemical methods [53, 54].

Various analytical methodologies have been developed for quantitative analysis of drugs in spiked human plasma [55–57]. Concerning the studied drugs, only one HPLC technique [22] was reported showing a lack of interference between LD and QUET but did not provide simultaneous quantitative determination of both drugs. To date, there has been no analytical method published in literature for simultaneously determining QUET, LD and CD. The aim of this work is to develop a white, simple, fast, reproducible and economical high-performance thin layer chromatographic (HPTLC) method for analyzing the studied mixture in human plasma samples, which could be applied in the future for therapeutic drug monitoring.

## Experimental

### Instrument and software

TLC Scanner (CAMAG model 3S/N 1302139) controlled with winCATS software for planar chromatography (CAMAG, Switzerland). Sample application was done in the form of bands using a 100.0 µL micro syringe inserted in CAMAG TLC autosampler Linomat (CAMAG, Muttenz, Switzerland). The band length is 6 mm, distance between bands equals 9.4 mm and application was done 10 mm from the plate’s bottom edge and its side edges. Aluminum HPTLC plates (20 × 10 cm) precoated with silica gel 60 F_254_ with 200 µm thickness (Merck, Germany).

### Chemicals and reagents

Pure QUET (purity100.0% ± 2.00%) was kindly provided by AstraZeneca UK Limited (Macclesfield, UK). Pure LD (purity 100.1% ± 2.00%) and CD (purity 99.5% ± 1.50%) were gratefully supplied from Divis Laboratories, India. Pure DOP (purity 99.8% ± 1.00%) was provided from Recordati (Milan, Italy). The purities of the four previously mentioned drugs were examined by applying official methods [17] for each drug and the results of assay percentages were found as written above. Methanol HPLC grade was purchased from Chem-Lab, Zedelgem, Belgium. All other reagents utilized were of analytical grade, including: anhydrous citric acid and acetone purchased from Chemajet, Cairo, Egypt; dichloromethane from Piochem, Giza, Egypt; glacial acetic acid and n-butanol from El-Nasr Pharmaceutical Chemical Co., Cairo, Egypt; and hydrochloric acid (HCl) from Sigma Aldrich, Darmstadt, Germany. Distilled water was used in mobile phase composition. The Holding Company of Biological Products and Vaccines (Vacsera) in Cairo, Egypt, provided the human plasma.

### Methods

#### Preparation of stock and working standard solutions

Separate stock standard solutions (each, 1.00 g/mL) for QUET and DOP (IS) were prepared using methanol. On the other hand, both stock standard solutions of LD and CD (each, 1.00 mg/mL) were prepared separately utilizing methanol containing 0.02 N HCl. Three sets of 5-mL measuring flasks were utilized to prepare working standard solutions for each of the three drugs (QUET, LD and CD). This was achieved by withdrawing appropriate aliquots from their corresponding stock solutions and completing the volumes to the mark by methanol. The concentration ranges for each drug in the prepared working standard solutions were: (2000–80,000 ng/mL), (4000–160,000 ng/mL) and (600–26,000 ng/mL) for QUET, LD and CD respectively.

#### Chromatographic conditions

HPTLC plates were dipped into 0.5% citric acid solution and left to dry in air overnight. Sample application (injection volume 100.0 µL) was done on the plates the following day. The developing system, which consists of acetone, dichloromethane, *n*-butanol, glacial acetic acid and water (3: 2.5: 2: 2: 1.75, by volume), was added to the chromatographic chamber, and saturation was done for 30 min at room temperature. Following that, ascending chromatography was performed to develop the plates for a distance 8.5 cm in a twin-trough chamber. After development, the plates were left air dried and subsequently UV scanning was done at 280 nm for the four selected drugs.

#### Spiking human plasma and sample pretreatment

Blank plasma equivalent to 425.0 µL was added to an Eppendorf tube. For each drug, 25.0 µL was withdrawn from its corresponding working standard solution, then added to blank plasma and mixed by vortex for 1 min. This resulted in a final volume of 500 µL of spiked plasma. The next step involved adding 50 µL of the working standard solution of the internal standard (DOP, 40 µg/mL) and vortex mixing again for 1 min. Protein precipitation technique was done by adding 1.5 mL of methanol containing 0.02 N HCl. Centrifugation was done at 14,000 rpm for 10 min at 5 °C. The clear supernatant was then withdrawn into a clean tube and evaporated under vacuum for 3 h. Reconstitution of the resulted residue was done in 0.5 mL methanol containing 0.02 N HCl and the general procedure was carried out as previously stated under Section “Chromatographic conditions”.

#### Preparation of calibrators

Calibrators were freshly prepared by withdrawing 25.0 µL of the appropriate working standard solution for each drug and spiking it into 425.0 µL of human blank plasma to reach a final volume of 500 µL. The final concentrations ranged from 100.0 to 4000.0 ng/mL for QUET, 200.0–8000.0 ng/mL for LD and 30.0–1300.0 ng/mL for CD. The same procedure of sample pretreatment was then followed as described under Section “Spiking human plasma and sample pretreatment”.

#### Quality control samples (QCs) preparation

During the bio-analytical validation, the quality control samples (QCs) were prepared at four concentration levels within the linear range to ensure accuracy and precision within and between analytical runs. The four levels are: Lower Limit of Quantification (LLOQ), low quality control (QCL) which is three times the LLOQ, medium quality control (QCM) which is 30–50% of the calibration range and finally high quality control (QCH) that is at least 75% of the upper Limit of Quantification (ULOQ). QCs were prepared in the same way as calibrators. QCs for QUET were: 100.0, 300.0, 1400.0 and 3200.0 ng/mL corresponding to LLOQ, QCL, QCM and QCH. As for LD, QCs were 200.0, 600.0, 2000.0 and 6000.0 ng/mL in accordance with LLOQ, QCL, QCM and QCH. Finally, QCs used for CD were 30.0, 60.0, 400.0 and 1000.0 relevant to LLOQ, QCL, QCM and QCH, respectively.

### Bioanalytical method validation

The proposed HPTLC methodology was validated according to US-FDA guidelines [58] for bioanalytical method validation.

#### Linearity

##### For pure standard solutions calibration

Eight concentration levels for each drug were utilized to construct calibration curve by plotting peak area ratios (integrated peak area of the analyte/peak area of IS) against the relevant concentration of standard solutions and the corresponding regression equations were calculated.

##### For in vitro calibration curve

Calibration curve was constructed using the following: eight concentration levels within the specified linearity range for each drug (each concentration level was applied in triplicates), LLOQ, a blank plasma sample and a zero calibrator (where internal standard was added to a blank plasma sample). Calibration curve was then achieved by plotting the peak area ratios against the corresponding concentrations for each of the three analytes (QUET, LD and CD) and the relevant regression equations were computed.

#### Accuracy and precision

For each of the three drugs (QUET, LD and CD), accuracy and inter- and intra-day precision were evaluated by analyzing five replicates of each of LLOQ and the three QCs (QCL, QCM and QCH) in at least three analytical runs over two consecutive days.

#### Selectivity

Blank plasma samples were injected to emphasize that they are free from interfering substances at the analytes’ retardation factor (R_f_).

#### Extraction recovery

Two sets of QC samples at their 3 levels (QCL, QCM and QCH) were prepared for each of the three studied drugs. The first set was prepared by spiking the plasma with drugs then applying extraction procedure. The second set was prepared by applying the extraction procedure first then spiking the drugs. The resulting average peak area was compared between the two sets for each concentration level and the percentage extraction recovery (% Ex. R) was calculated for each drug using the following equation:

$$ \% {\text{Ex}}. {\text{R}} = \frac{{{\text{Average}} {\text{peak}} {\text{area}} {\text{of}} {\text{pre}}\_{\text{extraction QC}} {\text{sample}} }}{{{\text{Average}} {\text{peak area of}} {\text{post}}\_{\text{extraction}} {\text{QC}} {\text{sample}}}} \times 100 $$

#### Stability

Short term stability (bench-top stability) was done by leaving QCL and QCH samples at room temperature (25 °C) for two hours then analyzing them in triplicate injection. On the other hand, three freeze–thaw cycles were done for both QCL and QCH, where samples were stored for at least 12 h at − 20 °C and then thawed. The cycle was repeated three times. Results of recoveries obtained were compared with those of fresh QC samples to calculate the deviation percentage (% Dev) using the equation below:

$$\% {\text{ Deviation}} = \frac{{\% {\text{R}} \,{\text{of}}\, {\text{old\, QC}} \,{\text{sample}} - \% {\text{R}}\, {\text{of}}\, {\text{fresh\, QC}} \,{\text{sample}}}}{{\% {\text{R}} \,{\text{of}}\, {\text{fresh\, QC}} \,{\text{sample}}}} \times 100$$

## Results and discussion

Whiteness assessment is a powerful approach that utilizes an integrated metric tool for calculating and comparing several analytical methods. A full assessment can be done by using the RGB-algorithm to evaluate the method’s reliability and implications. This enables the analyst to choose alternative analytical techniques that meet the criteria of higher analytical performance, greater greenness and increased cost and time effectiveness.

### Chromatographic conditions

A three solvent system composed of n-butanol, glacial acetic acid and water in various ratios was initially tried. Addition of organic solvents was essential to achieve the ideal separation of the studied drugs. These solvents include ethyl acetate, acetonitrile, acetone and others. The final system used as mobile phase was acetone, dichloromethane, n-butanol, glacial acetic acid and water (3: 2.5: 2: 2: 1.75, by volume). The problem of CD tailing was solved by predevelopment of HPTLC plates in 0.5% citric acid solution and leaving them to dry in air overnight (the day before sample application and plate development in mobile phase). All the above attempts were done to reach the most optimum separation and resolution between drugs. Consequently, system suitability parameters were computed [59, 60] revealing high selectivity, resolution and symmetric peaks as shown in Table 1. Scanning was done at several wavelengths, including: 248, 254, 280, 285 and 290 nm. The chosen wavelength was 280 nm at which all the four drugs showed sharp peaks and optimum absorbance.

**Table 1:** System suitability parameters of the proposed HPTLC-densitometric method for determination of QUET, LD and CD in presence of DOP in spiked plasma samples

	Calculated values for each drug in spiked plasma samples				Reference values [59, 60]
Parameter	LD	DOP	QUET	CD	
Retardation factor (R_f_) (± 0.02)	0.33	0.47	0.56	0.63	
Capacity factor (k´)	2.03	1.13	0.79	0.59	0–10
Selectivity (α) ^a^	1.80 1.43 1.34				α > 1
Resolution (R_s_) ^b^	2.15 1.64 1.56				R_s_ > 1.5
Tailing factor (T) ^c^	1.17	0.94	1.00	1.00	T ≈ 1 for a typical symmetric peak

^a^α = k´1 / k´2, where k´ is the capacity factor; k´ = (1 ‒ R_f_) / R_f_

^b^Rs = [2 (R_f__2_ ‒ R_f__1_)]/(W_1_ + W_2_), Where R_f__1_ and R_f__2_ are the retardation factors of two successive components; and W_1_ and W_2_ are the corresponding peak width at the peak base

^c^T = W_0.05_/2f, where W_0.05_ is the width of the peak at 5% height and f is the distance at 5% height from peak maximum to the leading edge of peak

### Spiking human plasma and sample pretreatment

For highly polar compounds such as LD and CD, liquid–liquid extraction is generally not the most effective option since these compounds predominantly partition and stay in the aqueous phase, resulting in limited recovery in the organic phase [61–63]. Consequently, protein precipitation technique was used. Different precipitating agents including ethanol, methanol and acetonitrile were tried. LD and CD showed better solubility in acidified aqueous solutions than organic solvents as previously discussed above. From this point, the precipitating agent used was methanol containing 0.02 N HCl. This revealed an improvement in drug extraction recoveries, for QUET, LD and CD as well as DOP.

### Bioanalytical method validation

QCs were injected in each analytical run to evaluate the validity and integrity of the results from the study samples analyzed in a single run as well as the performance of the developed bioanalytical method [58].

#### Linearity

**Fig. 2:**
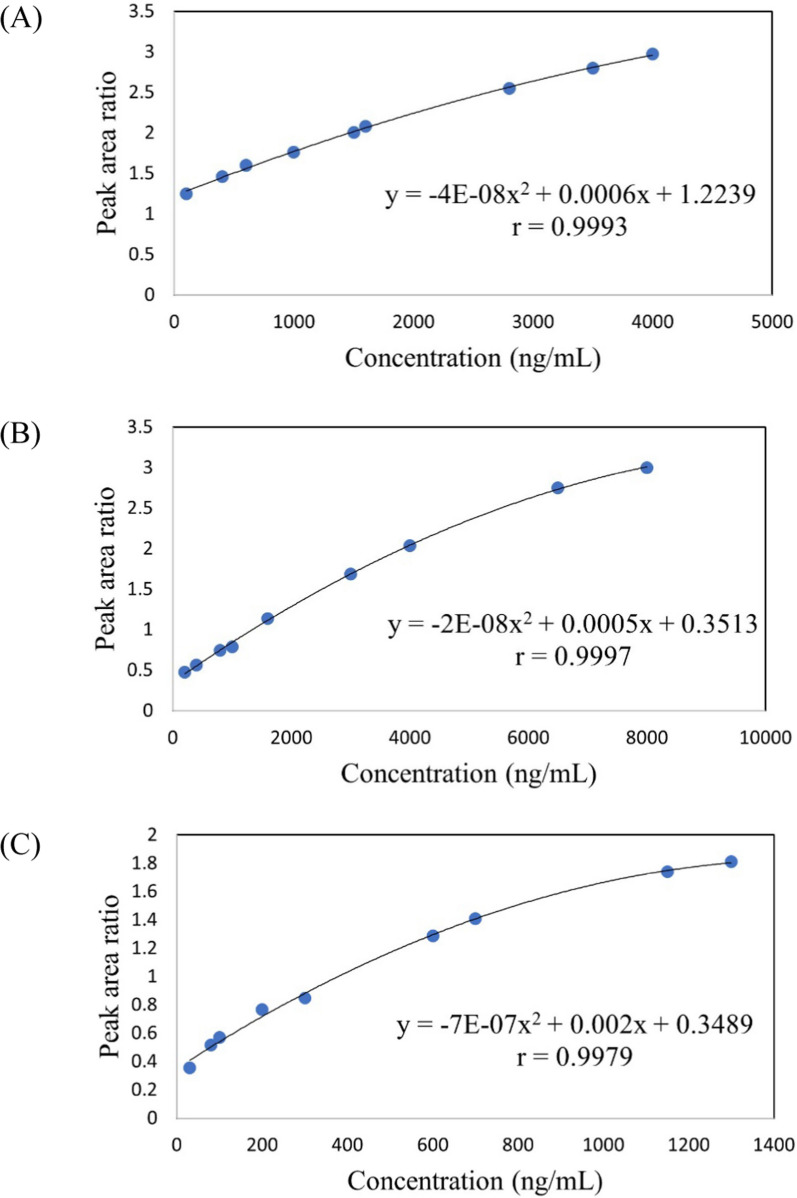
Calibration curves and relevant polynomial regression equations in spiked human plasma for (**A**) QUET, (**B**) LD and (**C**) CD

A calibration curve was constructed for each of the studied drugs following polynomial equation. The maximum plasma concentrations (C_max_) and therapeutic range were included in each calibration curve for each specified drug. Table 2 shows regression equation parameters for both pure standard solutions and spiked human plasma for QUET, LD and CD. The results of regression showed good correlation between peak area ratio and relevant concentrations for each specified drug as displayed in Fig. 2**.**

**Table 2:** Regression equation parameters of the proposed HPTLC-densitometric method for determination of QUET, LD and CD in pure standard solutions and spiked human plasma

Parameter	QUET	LD	CD	QUET	LD	CD
	Pure standard solution			Spiked human plasma samples		
Linearity range (ng/mL)	100–4000	100–8000	30–1300	100–4000	200–8000	30–1300
Regression model^a^	Polynomial	Polynomial	Polynomial	Polynomial	Polynomial	Polynomial
Coefficient 1 (b1)	− 4 × 10^–8^	− 3 × 10^–8^	− 7 × 10^–7^	− 4 × 10^–8^	− 2 × 10^–8^	− 7 × 10^–7^
Coefficient 2 (b2)	0.0002	0.0005	0.0018	0.0006	0.0005	0.002
Intercept (a)	0.7099	0.3928	0.2614	1.2239	0.3513	0.3489
Correlation coefficient (r)	0.9987	0.9991	0.999	0.9993	0.9997	0.9979

^a^Following a polynomial regression: A = b1C^2^ + b2C + a, where ‘A’ is the peak area ratio (peak area of analyte/ peak area of IS), ‘C’ is the concentration of QUET, LD and CD (ng/ml), ‘b1’ and ‘b2’ are coefficients 1 and 2, respectively and ‘a’ is the intercept

#### Accuracy and precision

Accuracy and precision were computed and were complying with the acceptable ranges according to FDA guidance. Where percentage recovery and percentage relative standard deviation (% RSD) are acceptable to be ± 15%, except for LLOQ which is allowed to be ± 20%. Accuracy, intra- and inter-day precision results are shown in Table 3.

**Table 3:** Intra- and Inter-day precision

Drug	Concentration (ng/mL)		Intra-day ^a^		Inter-day ^b^	
			Recovery (%)	% RSD	Recovery (%)	% RSD
QUET	LLOQ	100	99.78	0.16	97.41	3.10
	QCL	300	100.67	0.17	99.77	0.68
	QCM	1400	99.70	0.03	99.84	0.25
	QCH	3200	97.72	0.04	98.80	1.38
LD	LLOQ	200	97.56	1.17	98.53	2.41
	QCL	600	100.18	0.93	100.13	0.61
	QCM	2000	99.99	0.27	99.41	1.05
	QCH	6000	100.29	0.09	100.10	0.18
CD	LLOQ	30	95.68	0.97	92.22	4.30
	QCL	60	98.47	0.50	97.69	0.84
	QCM	400	99.47	0.12	98.83	1.28
	QCH	1000	100.54	1.47	100.79	1.28

^a^ n = 5

^b^ n = 15

#### Selectivity

**Fig. 3:**
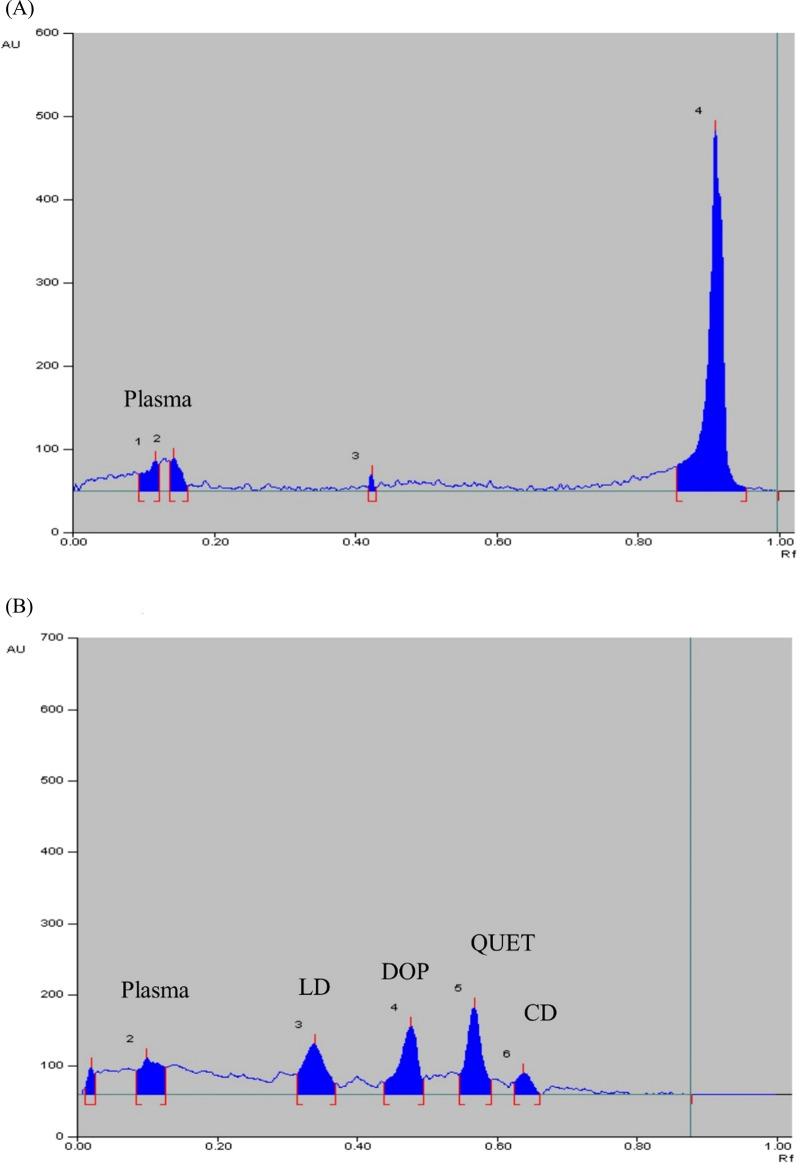
HPTLC-densitogram of (**A**) Blank human plasma, (**B**) Human plasma spiked with QUET (120 ng/mL), LD (900 ng/mL) and CD (45 ng/mL) in the presence of DOP (IS) (4000 ng/mL) at their Cmax

Selectivity is defined as being able to identify and quantitatively determine the studied drugs without being impacted by endogenous or other compounds present in plasma. The method proved to be selective by injecting blank plasma samples that showed no interfering peak at the R_f_ values of the four drugs as seen in Fig. 3.

#### Sensitivity

LLOQ was determined by signal to noise ratio which is more than 10. LLOQ values were 100.0, 200.0 and 30.0 ng/mL for QUET, LD and CD respectively.

#### Extraction recovery

After the analysis of QC samples, % Ex. R and % RSD were calculated for the average of three determinations at each QC level for each drug, as shown in Table 4**.** The table reveals good extraction recoveries extending from 92.89% to 99.32% with optimum reproducibility where % RSD lies between 0.53% and 5.52%. This proves the successful performance of the method for the bioanalysis of the studied drugs.

**Table 4:** Results of extraction recovery percentage for QUET, LD and CD in spiked human plasma

Drug	Concentration (ng/mL)		Recovery (%)^a^	% RSD^a^
QUET	QCL	300	99.32	1.00
	QCM	1400	97.38	4.84
	QCH	3200	94.98	0.87
LD	QCL	600	94.27	3.89
	QCM	2000	92.89	5.52
	QCH	6000	96.74	1.94
CD	QCL	60	95.92	0.83
	QCM	400	97.43	0.53
	QCH	1000	98.29	1.11

^a^ n = 3

#### Stability

Benchtop (short term stability) and freeze thaw stability were performed and results of % recovery, % RSD and % Dev are presented in Table 5. QC samples were regarded stable under the investigated stability conditions where they complied with the acceptable limit for % recovery which is ± 15%.

**Table 5:** Results of stability of QUET, LD and CD in spiked human plasma

Drug	Concentration (ng/mL)		Benchtop stability			Freeze and thaw stability		
			Recovery (%)^a^	% RSD^a^	% Dev^b^	Recovery (%)^a^	% RSD^a^	% Dev^b^
QUET	QCL	300.0	98.76	0.45	− 1.97	96.68	0.91	− 4.03
	QCH	3200.0	97.44	0.08	− 3.04	99.46	1.63	− 1.04
LD	QCL	600.0	99.13	0.70	− 1.11	98.34	1.44	− 1.90
	QCH	6000.0	97.91	1.57	− 2.00	102.23	7.00	2.29
CD	QCL	60.0	97.05	5.28	− 1.47	94.90	3.48	− 3.68
	QCH	1000.0	96.80	2.44	− 2.69	96.37	1.66	− 3.13

^a^ n = 3

^b^ % Dev = [(% R of old QC sample—% R of fresh QC sample) / % R of fresh QC sample] × 100

### Prescribed doses and relevant plasma concentration levels of the studied drugs in case of PDP

In literature, it is reported that the target therapeutic dose of QUET in case of PDP ranges from 50 to 150 mg/day [11, 14, 64] according to the patient’s management case. These doses are lower than those prescribed for schizophrenia (300–800 mg/day) [65]. Previously reported pharmacokinetic studies for quetiapine in human volunteers showed the relevance of the quetiapine doses on its concentration levels in plasma after 1.5 to 2 h of administration (the time to reach C_max_) [18, 23, 66]. It was concluded that plasma concentration level reached were ranging from 100 to 500 ng/mL according to the prescribed dose (50–150 mg/day). On the other hand, C_max_ for LD and CD were stated in therapeutic ranges 500–1600 and 40–225 ng/mL according to the prescribed doses [67].

### Comparison between the proposed method and the previously reported methods

HPTLC offers a greener alternative compared to other chromatographic techniques specially HPLC [68]. Consuming just few microliters of the sample were enough for identification and quantitative determination of the analyte of interest. On the other hand, 10 mL or more would be yielded as analytical waste for the same reading of the same analyte on HPLC. Furthermore, when it comes to the overall operating expenses of the analytical method, which include the price of the instrument, detector, column, solvents of high purity, power consumption, and procedures for sample purification, HPTLC is preferably chosen over HPLC from the economical point of view [69]. Neither a complex instrument nor a high-power supply is needed during the entire process of separation. Additional advantage of HPTLC is its ability to run several samples simultaneously instead of sequentially, allowing spotting of 20 samples on a small plate (20 × 10 cm) and running under the same experimental conditions [70]. However, in HPLC, samples must be injected one after the other on a column that has been pre-washed and pre-conditioned which certainly causes a delay in acquisition of data [71]. From another perspective, spectrofluorimetric methods essentially require derivatization procedure which has high impact on environment and lowers the greenness score in green analytical chemistry metrics. As for electrochemical methods, electrode modification is generally essential to reach plasma concentration levels of the studied drugs and this could be expensive and time consuming in some cases. Additional file 1: Table S1 shows the linearity ranges, detection limits and quantitation limits for previously reported analytical methods using various techniques and different matrices including human plasma and rat plasma. It is concluded that the proposed method achieved linearity ranges near to the previously reported ones for the three drugs QUET, LD and CD especially for methods using UV-detectors. The simplicity of application of the suggested analytical method together with its good results achieved in bioanalytical validation makes it reliable for future analysis of the three aforementioned drugs in biological samples.

### Greenness evaluation

Greenness assessment tools were used to appraise the ecological sustainability of the suggested analytical method in comparison with two reported HPLC methods [18, 39].

#### Green solvent selection tool

**Fig. 4:**
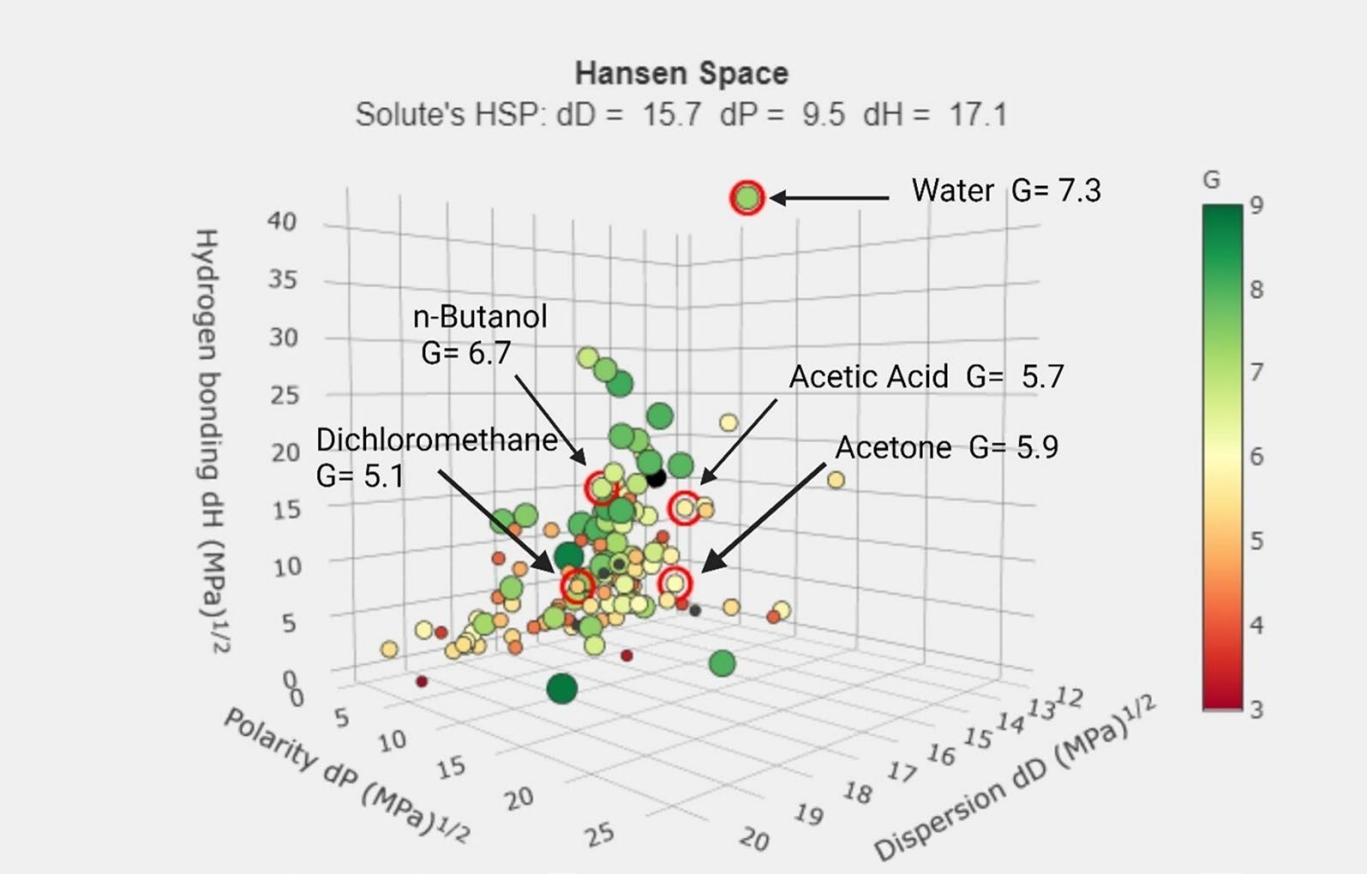
G-scores of the solvents used in mobile phase

An online application tool for identifying green solvents according to GlaxoSmithKline (GSK) Solvent Sustainability Guidelines [6, 72]. It uses a composite score that takes into account many parameters to provide a numerical assessment of solvents, mainly depending on Hansen Solubility Parameter (HSP) during computation. Three primary parameters are considered: dispersion forces (dD), polar forces (dP), and hydrogen bonding forces (dH). The G-scores for the solvents used is presented in Fig. 4**.** The higher the G-score value, the more sustainable the solvent used.

##### Analytical Eco-scale

Eco-scale is one of the most effective semi-quantitative tools for comparing and selecting the most environmentally friendly method for analysis. It relies on computing penalty points for a group of factors including: reagents utilized, energy consumption, risk associated with workplace and waste generation. The total calculated penalty points are then subtracted from 100 and the resulting score evaluates the level of greenness of the method. The proposed method achieved higher analytical Eco-scale score in comparison with the two reported HPLC methods as demonstrated in Table 6.

**Table 6:** Calculated penalty points (PPs) for the proposed and reported methods using Analytical Eco-scale

Parameters	Penalty points (PPs)		
	Proposed HPTLC Method	Reported HPLC Method^a^ [18]	Reported HPLC Method^b^ [39]
Reagents			
Methanol	6	12	12
Phosphate buffer	–	3	3
Acetonitrile	–	8	–
TEA	–	6	–
HCl	4	–	4
Acetone	4	–	–
Dichloromethane	2	–	–
n-Butanol	6	–	–
Glacial acetic acid	4	–	–
Water	0	0	0
Instrument			
Energy	0 (≤ 0.1 kWh per sample)	1 (≤ 1.5 kWh per sample)	1 (≤ 1.5 kWh per sample)
Occupational hazard	0	3	3
Waste	1	5	5
Total PPs	Ʃ 24	Ʃ 38	Ʃ 28
Analytical Eco-scale score^c^	76	62	72

^a^Reported HPLC method for determination of Quetiapine

^b^Reported HPLC method for determination of Levodopa and Carbidopa

^c^Analytical eco-scale score = 100 (the ideal score for green analytical method)

^c^Analytical eco-scale score > 75 (a great green analysis)

^c^Analytical eco-scale score 50–75 (green analysis is acceptable)

^c^Analytical eco-scale score < 50 (green analysis is inadequate)

##### Green analytical procedure index

GAPI is regarded as an innovative approach that could be effectively employed to assess the greenness of an analytical method. It consists of fifteen elements which evaluate the steps done throughout the entire analysis and are represented in five pentagrams. Based on the GAPI color system, red color refers to a substantial environmental risk, yellow indicates lesser environmental risk and green represent eco-friendliness of the analytical method. More green colored areas reflect more eco-friendliness and lower environmental impact. The proposed method showed seven green pictograms and only three red ones. On the other hand, the two reported HPLC methods showed five green pictograms and five red ones as displayed in Table 7.

**Table 7:** Comparison between the proposed HPTLC and the reported methods in terms of greenness assessment using GAPI and AGREE tools

Chromatographic condition	GAPI	AGREE^*^
Proposed HPTLC method Chromatographic conditions: Aluminum HPTLC plates precoated with silica gel 60 F254 as stationary phase, mobile phase consisted of Acetone, Dichloromethane, n-Butanol, Glacial acetic acid and Water (3: 2.5: 2: 2: 1.75, by volume) and UV detection was done at 280 nm		
Reported HPLC method [18] Chromatographic conditions: reversed phase Nova pack C18 column as stationary phase, mobile phase consisted of acetonitrile, methanol and 0.025 M phosphate buffer (Containing 1 mL TEA in each 250 mL, pH was adjusted to 5.5 with 0.2 M phosphoric acid) in a ratio of (40: 30: 30%, by volume). Flow rate at 1.2 mL/min and UV detection was performed at 225 nm		
Reported HPLC method [39] Chromatographic conditions: Hypersil-ODS column as stationary phase, mobile phase consisted of methanol and 0.002 M KH2PO4 (pH 5) solution in a ratio of (25: 75%, v/v). Flow rate at 1 mL/min and column temperature was adjusted at 30 °C. Diode array detector was used and photometric detection was done in the range of 190–400 nm		

##### Analytical greenness metric approach

An analytical greenness metric tool was utilized. It computes the twelve GAC parameters. For each parameter, a scale from 0 to 1 is generated. The outcome is a numerical value found in the central area of pictogram which ranges between 0 and 1 according to the method’s level of greenness. If the achieved score is near to 1, the method is considered green. The proposed analytical method complies with the majority of twelve principles of GAC. No additional sample pretreatment steps or derivatization were included. Minimal sample size was used and minimal volume of analytical waste was produced; since twin-trough chamber was used which consumes approximately 10 to 15 mL only of mobile phase [73]. Furthermore, the developed method quantifies multiple analytes which is more preferred than methods using a single analyte at a time. Acetone, n-butanol, acetic acid and water constitute a significant portion of the mobile phase. These solvents are derived from renewable sources [74]. Table 7 shows that higher score is achieved by the proposed method owing to the previously discussed reasons.

### Whiteness evaluation using the RGB-algorithm method

Further assessment of the proposed method’s environmental impact has been carried out. WAC focuses on several features of the analytical technique, such as its practicality, affordability and influence on the environment. Each feature is displayed by a color; where red expresses analytical performance, green indicates safety and eco-friendliness and blue refers to productivity and practical effectiveness. The combination of the three colors generates the white color which is the ideal case. In other situations, the three colors may be combined in uneven proportions giving rise to a different color rather than white (ranging from black to white). Upon applying RGB- algorithm for the proposed method, the final score achieved is 82% and the method color is white as found in Table 8. This proves the reliability of the proposed method in many aspects including greenness, analytical performance and cost effectiveness. On the other hand, the two reported HPLC methods [18, 39] reached final scores of 63.7% and 62.8%, respectively. The color of both methods is red, which reflects high analytical performance but a low level of greenness and productivity.

**Table 8:**
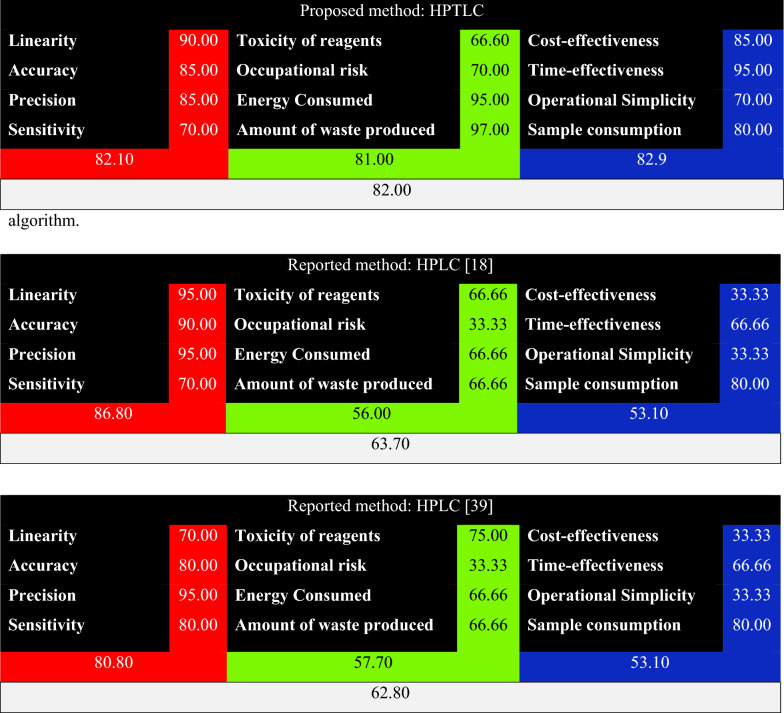
Whiteness assessment of the proposed HPTLC method and two reported HPLC methods using RGB-algorithm

## Conclusion

Developing analytical procedures for quantifying drugs in biological matrices is of great importance; especially when it comes to patients taking multiple medications as in case of Parkinson’s disease patients. The proposed analytical method proved to be white, simple, low cost, selective, fast and sustainable. Successful application to spiked human plasma and high extraction recoveries for QUET, LD and CD were achieved with optimum reproducibility. This enables the practical application of the developed method in future therapeutic drug monitoring studies. Furthermore, green analytical metrics and whiteness appraisal were complementary to each other to provide a full assessment of greenness and efficiency of the proposed method.

## Supplementary Information

Additional file 1.

## Data Availability

The datasets used and/or analysed during the current study are available from the corresponding author on reasonable request.

## Abbreviations

AGREE: Analytical greenness metric approach
CD: Carbidopa
C_max_: Maximum plasma concentrations
CZE: Capillary zone electrophoresis
DOP: Dopamine
dD: Dispersion forces
dP: Polar forces
dH: Hydrogen bonding forces
GAC: Green analytical chemistry
GAPI: Green analytical procedure index
GC–MS/MS: Gas Chromatography with tandem mass spectrometry
GSK: GlaxoSmithKline
HPTLC: High performance thin layer chromatography
HPLC: High performance liquid chromatography
HCl: Hydrochloric acid
HSP: Hansen solubility parameter
IS: Internal standard
LD: Levodopa
LC–MS/MS: Liquid Chromatography with tandem mass spectrometry
LLOQ: Lower limit of quantification
PDP: Parkinson’s disease psychosis
PD: Parkinson’s disease
QUET: Quetiapine
QCs: Quality control samples
QCL: Low quality control
QCM: Medium quality control
QCH: High quality control
RGB: Red–Green–Blue
TLC: Thin layer chromatography
USP: United States Pharmacopeia
ULOQ: Upper limit of quantification
Vacsera: The holding company of biological products and vaccines
WAC: White analytical chemistry
% Dev: Deviation percentage
% Ex. R: Percentage extraction recovery
% RSD: Percentage relative standard deviation
