# Supplementary material for: Greenness and whiteness appraisal for bioanalysis of quetiapine, levodopa and carbidopa in spiked human plasma by high performance thin layer chromatography
Source: BMC Chem. 2024 Oct 21;18(1):205. doi: 10.1186/s13065-024-01309-w (PMC11495128; doi:10.1186/s13065-024-01309-w)
Supplement: Supplementary file 1 — Additional file 1. [file 13065_2024_1309_MOESM1_ESM.docx]

**Table S1:**  Comparison of previously reported methods for the determination of QUET, LD and CD with the present work.

| Analytical Technique | Analyte | Linearity range  (ng/mL) | LLOQ  (ng/mL) | LOD  (ng/mL) | Application | Refs. |
| --- | --- | --- | --- | --- | --- | --- |
| HPLC-UV | QUET | 80-2000 | 80 | 30 | Pharmaceutical formulation Human plasma | [1] |
| HPLC-UV | QUET | 4-400 | 4 | 1.5 | Human plasma | [2] |
| HPLC (UV and electrochemical detection) | QUET | 2.5-500 | ─ | ─ | Human plasma | [3] |
| HPLC- UV | QUET | 200-3000 | 190 | 30 | Human plasma | [4] |
| TLC-UV  HPLC-UV | QUET | 150-3000  150-5000 | 150  150 | ─ | Rat plasma | [5] |
| HPLC-UV | QUET | 70-9000 | 50 | 13 | Human plasma | [6] |
| Spectrofluorimetry | QUET | 200-2000 | 170 | 50 | Pharmaceutical formulation  Human plasma | [7] |
| Spectrofluorimetric | QUET | 20-1000 | 20 | 6.3 | Pharmaceutical formulation  Rat plasma | [8] |
| LC-MS/MS | QUET | 1-1000 | 1 | ─ | Human serum | [9] |
| GC-MS/MS | QUET | 40-600 | 40 | 10 | Human plasma | [10] |
| Potentiometry | QUET | 880- 88×10^5^  for sensor Ι  88- 88×10^5^  for sensor ΙΙ | ─ | 1584  For sensor Ι 176  For sensor ΙΙ | Human plasma | [11] |
| Voltammetry | QUET | 5298.60- 176620 | 1792.69 | 536.92 | Human serum Urine | [12] |
| HPLC-UV | LD  CD | 25-1200  50-1000 | 30  20 | 10  6.16 | Pharmaceutical formulation  Human plasma | [13] |
| HPLC-DAD | LD  CD | 0-7000  0-6000 | 161  24 | 53.10  7.90 | Human plasma | [14] |
| HPLC-UV | LD | 100-10000 | 100 | 25 | Human plasma | [15] |
| HPLC (electrochemical detection) | LD  CD | 25-5000  25-1000 | 25  25 | ─ | Human plasma | [16] |
| HPLC (electrochemical detection) | LD  CD | 10-2000  10-2000 | 10  10 | ─ | Rat plasma | [17] |
| kinetic-spectrophotometric | LD  CD | 200-6000  200-7000 | 200  200 | 120  140 | Pharmaceutical formulation  Human serum | [18] |
| Spectrofluorimetric | LD | 19.72-985.95 | 19.72 | 1.91 | Human serum Urine | [19] |
| LC-MS/MS | LD  CD | 20-1000  30-600 | 20  30 | 5  7 | Human plasma  Pharmaceutical formulation | [20] |
| Voltammetry | LD  CD | 394.38- 5324.13  452.46-5203.29 | ─ | 165.64  147.05 | Pharmaceutical formulation  Human serum | [21] |
| HPTLC- UV | QUET  LD  CD | 100-4000  200-8000  30-1300 | 100  200  30 | 50  150  25 | Human plasma | This  work |

**References**

1. Belal F, Elbrashy A, Eid M, Nasr JJ. Stability-Indicating HPLC Method for the Determination of Quetiapine: Application to Tablets and Human Plasma. J Liq Chromatogr Relat Technol. 2008;31:1283–98.

2. Mandrioli R, Fanali S, Ferranti A, Raggi MA. HPLC analysis of the novel antipsychotic drug quetiapine in human plasma. J Pharm Biomed Anal. 2002;30:969–77.

3. Davis PC, Wong J, Gefvert O. Analysis and pharmacokinetics of quetiapine and two metabolites in human plasma using reversed-phase HPLC with ultraviolet and electrochemical detection. J Pharm Biomed Anal. 1999;20:271–82.

4. Youssef RM, Abdine HH, Barary MA, Wagih MM. Selective RP-HPLC Method for Determination of Quetiapine in Presence of Coadministered Drugs: Application for Long-Term Stability Study of Quetiapine in Whole Blood. Acta Chromatographica. 2016;28:263–79.

5. Abdelwahab NS, Ahmed AB, Omar MA, Derayea SM, Abdelrahman MM. Green chromatographic methods for simultaneous determination of quetiapine and the co-administrated paroxetine in rat plasma with application to pharmacokinetic study. Microchemical Journal. 2020;152.

6. Li D, Zou J, Cai P-S, Xiong C-M, Ruan J-L. Preparation of magnetic ODS-PAN thin-films for microextraction of quetiapine and clozapine in plasma and urine samples followed by HPLC-UV detection. J Pharm Biomed Anal. 2016;125:319–28.

7. Mostafa IM, Omar MA, Nagy DM, Derayea SM. Analysis of quetiapine in human plasma using fluorescence spectroscopy. Spectrochim Acta A Mol Biomol Spectrosc. 2018;196:196–201.

8. Derayea SM, Ahmed AB, Abdelwahab NS, Abdelrahman MM, Omar MA. Innovative spectrofluorometric protocol based on micro-environment improvement for determination of Quetiapine in dosage forms and rat plasma. Spectrochim Acta A Mol Biomol Spectrosc. 2020;233.

9. Miroshnichenko II, Baymeeva N V. Simultaneous Determination of Antipsychotic Drugs and Their Active Metabolites by LC-MS-MS and its Application to Therapeutic Drug Monitoring. J Chromatogr Sci. 2018;56:510–7.

10. Rosado T, Oppolzer D, Cruz B, Barroso M, Varela S, Oliveira V, et al. Development and validation of GC/MS/MS method for simultaneous quantitation of several antipsychotics in human plasma and oral fluid. Rapid Communication in Mass Spectrometry. 2018;32.

11. Nebsen M, El-Maraghy C, Salem H, Amer S. Novel Membrane Sensors for the Determination of Quetiapine Fumarate in Plasma and in Presence of its Related Compounds. Anal Bioanal Electrochem. 2016;8:51–63.

12. Ozkan SA, Dogan B, Uslu B. Voltammetric Analysis of the Novel Atypical Antipsychotic Drug Quetiapine in Human Serum and Urine. Microchim Acta. 2006;153:27–35.

13. Belal F, Ibrahim F, Sheribah ZA, Alaa H. Micellar HPLC-UV method for the simultaneous determination of levodopa, carbidopa and entacapone in pharmaceuticals and human plasma. J Chromatogr B Analyt Technol Biomed Life Sci. 2018;1091:36–45.

14. Li S-F, Wu H-L, Yu Y-J, Li Y-N, Nie J-F, Fu H-Y, et al. Quantitative analysis of levodopa, carbidopa and methyldopa in human plasma samples using HPLC-DAD combined with second-order calibration based on alternating trilinear decomposition algorithm. Talanta. 2010;81:805–12.

15. Elbarbry F, Nguyen V, Mirka A, Zwickey H, Rosenbaum R. A New Validated HPLC Method for the Determination of Levodopa: Application to Study the impact of ketogenic diet on the Pharmacokinetics of Levodopa in Parkinson’s Participants. Biomed Chromatogr. 2019;33.

16. Titus DC, August TF, Yeh KC, Eisenhandler R, Bayne WF, Musson DG. Simultaneous high-performance liquid chromatographic analysis of carbidopa, levodopa and 3-0-methyldopa in plasma and carbidopa, levodopa and dopamine in urine using electrochemical detection. J Chromatogr. 1990;534:87–100.

17. Raut PP, Charde Y, Bishnoi P. Simultaneous estimation of levodopa, carbidopa and 3-oxymethyldopa in rat plasma using HPLC-ECD. Biomed Chromatogr. 2016;30:1696–7000.

18. Chamsaz M, Safavi A, Fadaee J. Simultaneous kinetic-spectrophotometric determination of carbidopa, levodopa and methyldopa in the presence of citrate with the aid of multivariate calibration and artificial neural networks. Anal Chim Acta. 2007;603:140–6.

19. An J, Shi Y, Fang J, Hu Y, Liu Y. Multichannel ratiometric fluorescence sensor arrays for rapid visual monitoring of epinephrine, norepinephrine, and levodopa. Chemical Engineering Journal. 2021;425.

20. Ribeiro RP, Gasparetto JC, de Oliveira Vilhena R, de Francisco TMG, Martins CAF, Cardoso MA, et al. Simultaneous determination of levodopa, carbidopa, entacapone, tolcapone, 3- O -methyldopa and dopamine in human plasma by an HPLC–MS/MS method. Bioanalysis. 2015;7:207–20.

21. Hissashi Takeda H, Almeida Silva T, Campos Janegitz B, Campanhã Vicentini F, Henrique Capparelli Mattoso L, Fatibello-Filho O. Electrochemical sensing of levodopa or carbidopa using a glassy carbon electrode modified with carbon nanotubes within a poly(allylamine hydrochloride) film. The Royal Society of Chemistry. 2016;:1274–80.
